# Increased Expression of CC16 in Patients with Idiopathic Pulmonary Fibrosis

**DOI:** 10.1371/journal.pone.0168552

**Published:** 2016-12-15

**Authors:** Ivette Buendía-Roldán, Víctor Ruiz, Patricia Sierra, Eduardo Montes, Remedios Ramírez, Anita Vega, Alfonso Salgado, Mario H. Vargas, Mayra Mejía, Annie Pardo, Moisés Selman

**Affiliations:** 1 Instituto Nacional de Enfermedades Respiratorias Ismael Cosío Villegas, México City, México; 2 Facultad de Ciencias, Universidad Nacional Autónoma de México, México City, México; University of Giessen Lung Center, GERMANY

## Abstract

Idiopathic pulmonary fibrosis (IPF) is a devastating disease of unknown etiology. The pathogenic mechanisms are unclear, but evidence indicates that aberrantly activated alveolar epithelial cells secrete a variety of mediators which induce the migration, proliferation and activation of fibroblasts and finally the excessive accumulation of extracellular matrix with the consequent destruction of the lung parenchyma. CC16 (approved symbol SCGB1A1), a putative anti-inflammatory protein produced by “club” cells in the distal airways, has not been evaluated in IPF lungs. In this study, we determined the serum and bronchoalveolar lavage (BAL) levels as well as the lung cell localization of this protein. Also, we explored the usefulness of serum levels of CC16 for the differential diagnosis of IPF (n = 85), compared with non-IPF interstitial lung diseases [chronic hypersensitivity pneumonitis (cHP; n = 85) and connective tissue diseases (CTD-ILD; n = 85)]. CC16 was significantly increased in serum and BAL fluids of IPF patients and was found not only in club cells but also in alveolar epithelial cells. When compared with non-IPF patients and controls, serum levels were significantly increased (p<0.0001). Sensitivity and specificity for CC16 (cut-off 41ng/mL) were 24% and 90%, positive predictive value 56% and negative predictive value 69%. These findings demonstrate that CC16 is upregulated in IPF patients suggesting that may participate in its pathogenesis. Although higher than the serum levels of non-IPF patients it shows modest sensitivity to be useful as a potential biomarker for the differential diagnosis.

## Introduction

Idiopathic pulmonary fibrosis (IPF) is a chronic, progressive, and usually lethal fibrosing interstitial pneumonia of unknown etiology and a median survival of 2–3 years after diagnosis [1]. IPF occurs primarily in older adults and is diagnosed based in the finding of usual interstitial pneumonia (UIP) proven by histopathology and/or high resolution computed tomography (HRCT) [1].

The pathogenic mechanisms have not been elucidated, but a growing body of evidence indicates bronchoalveolar epithelial cells play a key role in the initiation and perpetuation of the disease. These epithelial cells are highly active and secrete a variety of growth factors, cytokines, chemokines, matrix metalloproteinases and coagulation factors that participate in the formation of the fibroblastic/myofibroblastic foci and the subsequent abnormal tissue remodeling [2]. In this context, we aimed to evaluate Club cell protein 16 (CC16) a mediator produced by non-ciliated airway epithelium, primarily bronchiolar club cells, with putative anti-inflammatory properties. CC16 has been found decreased in smoke-exposed lungs, and chronic obstructive pulmonary disease (COPD), but studies in IPF are scanty [3]. Interestingly, there is evidence that club cells may induce apoptosis of alveolar and bronchiolar epithelial cells, a process that play a critical role in the pathogenesis of IPF [4].

In addition, we aimed to determine whether serum concentration of CC16 may distinguish IPF patients from those with some non-IPF interstitial lung diseases (ILD) that may also present an UIP-like pattern. Actually, it is well known that a UIP-like pattern may be found in other fibrotic lung diseases mainly in some connective tissue-diseases (CTD-ILD) [5, 6] and chronic hypersensitivity pneumonitis (cHP) [7, 8]. The accurate distinction between IPF and other fibrotic lung disorders is extremely important because there are some recently described therapeutic agents which target key pro-fibrotic signaling pathways and have been shown to be effective specifically in IPF [9, 10]. By contrast, the other inflammatory-driven diseases are treated with anti-inflammatory and immunosuppressive drugs that are deleterious in IPF increasing the risk of hospitalization and death [11].

A variety of biomarkers related to alveolar epithelial cell dysfunction or fibroproliferation and matrix deposition has been described in IPF [12, 13], but in general, they seem to be useful as predictive tools and efforts toward identifying biomarkers that may help to the differential diagnosis are scanty. Recently, a biomarker index composed by SP-D, MMP-7, and osteopontin showed to improve diagnostic accuracy of IPF compared to non-IPF ILD [14].

In this context, the aim of this study was to assess the expression and lung localization of CC16 in IPF lungs and its diagnostic value to distinguish IPF from non-IPF patients.

## Materials and Methods

### Study population

The concentration of CC16 in serum and bronchoalveolar lavage fluids was performed using samples already obtained and maintained at -70°C from a population of IPF and non-IPF patients attending the Interstitial Lung Disease Clinic of the National Institute of Respiratory Diseases in the last 8 years. Our serum bank comprises 502 patients with IPF, 651 patients with cHP, and 287 patients with CTD-ILD. From this population, 85 patients from each group were randomly selected for quantification of serum CC16. In the CTD-ILD group, only patients with rheumatoid arthritis or Sjögren syndrome were included. Diagnosis of each disease was performed according to accepted international criteria [1, 15–17]. Patients with IPF did not display either airways disease or emphysematous lesions and were stable for at list three months before serum was taken. All patients signed an informed consent letter authorizing the use of their serum and BAL fluids in future studies. The control group was integrated by 30 subjects of our “Aging Lung Program” without pulmonary diseases, as assessed by the lack of symptoms, normal spirometry and computed tomography, and who were matched by IPF age. All these subjects signed a consent letter. In addition, the levels of CC16 in bronchoalveolar lavage fluids (BALF) were determined in 58 IPF and 8 controls. The protocol was approved by the BioEthic Committee of INER (approval number C05-13).

Demographic information was obtained from all patients (age, sex), medical history (time of symptoms before diagnosis, co-morbidities, including treatment and duration, family history of lung disease), physical examination, respiratory function tests (spirometry, plethysmography, carbon monoxide diffusion, walking distance (meters) and changes in oxygen saturation in the 6-minute walk).

### Analysis of serum CC16

All blood samples were obtained on fasting and after at least 10 minutes rest, to control the postural effects on blood measurements. Samples were aliquoted and frozen, and thawed only once before measurement CC16. The quantification of CC16 was performed by ELISA, by duplicate, according to the manufacturer's instructions using the Human Cell Protein Club kit, (BioVendor, Karasek, Czech Republic).

### Bronchoalveolar lavage

As part of the diagnostic process, bronchoalveolar lavage (BAL) was achieved as described [18]. Briefly, BAL was performed through flexible fiberoptic bronchoscopy under local anesthesia and 300 ml of normal saline were instilled in 50-ml aliquots, with an average recovery of 60%–70%. The recovered BAL fluid was centrifuged at 250 g for 10 min at 4°C and the supernatants were kept at −70°C until use.

### Immunohistochemistry

Cell localization of CC16 was performed in lung biopsies of IPF patients and controls tissues as previously described [19]. Briefly, sections of tissue blocks of formalin-fixed lung samples were incubated at 4°C overnight with the specific antibody for detection of CCL16 (anti-Clara Cell Protein Secretory Antibody, Millipore cat 07–623 Merck KGaA, Darmstadt, Germany). A secondary biotinylated anti-IgG followed by horseradish peroxidase–conjugated streptavidin (BioGenex, San Ramon, CA) was used according to manufacturer’s instructions. 3-amino-9-ethyl-carbazole in acetate buffer with 0.05% H_2_O_2_ was used as substrate. The experiments included controls in which the primary or secondary antibody was omitted.

### Immuno-colocalization of CC16 and surfactant protein C

Immunofluorescence staining was performed in 3μm thick tissue sections adhered to silanized slides. After heat-induced antigen retrieval, samples were permeabilized and blocked in a single step incubating them for 30 minutes in PBS with 2% normal pig serum and 0.5% Triton X-100 (Research Organic, Cleveland, OH, USA) at room temperature (RT). Afterwards samples were washed and incubated at 4°C overnight with the primary antibodies: anti-CC16 (anti-Clara Cell Protein Secretory Antibody, Millipore cat 07–623 Merck KGaA, Darmstadt, Germany) and anti-prosurfactant Protein C (ab40879, Abcam, Cambridge, UK). After incubation, samples were washed and incubated with fluorescent secondary antibodies (Alexa Fluor 546 conjugated Donkey Anti-Mouse IgG and Alexa Fluor 647 conjugated Donkey Anti-Rabbit IgG; Jackson Immunoresearch, West Grove, PA) for 1 hour at RT. Samples were mounted with ProLong Gold mounting media (Life Technologies). All experiments included controls with/without primary antibody or with/without secondary antibody. Image capture was done with an FV-1000 confocal laser scanning microscope (Olympus Corporation, Tokyo, Japan) in sequential scanning mode to image each fluorochrome individually.

### Statistical analysis

Results were collected in Excel and analyzed in the Stata statistical package, version 11. Results are expressed as mean with standard deviations. An exploratory data analysis was performed to identify inconsistent data entry errors. Univariate analysis of each variable was done to calculate the measures of central tendency and dispersion of continuous variables and proportions for categorical variables. In bivariate analysis, mean differences and proportions according to each group were calculated and Mann-Whitney U test and ANOVA were applied. We defined p value <0.05 as statistical significant difference. Receiver operating characteristic (ROC) curve was generated to determine the sensitivity threshold (Y axis) and 1-specificity (X axis) of CC16. The area under the curve (AUC) was used to compare and determine the power of discrimination of CC16 to diagnose IPF. Likewise the positive and negative predictive values of serum CC16 as biomarker were determined. Multivariate linear regression model and Spearman correlation was used to assess the association between CC16 (dependent variable) and relevant demographic and functional independent variables (gender, smoking, FVC, and DLCO and oxygen saturation at rest).

## Results

### Demographic profile

Eighty five patients with IPF, 170 non-IPF patients (85 cHP and 85 CTD-ILD), and 30 healthy individuals were included in this study. Demographic characteristics and lung function data are shown in Table 1. Patients with cHP and CTD-ILD were younger than IPF and controls, but the difference was not statistically significant. There was female predominance in cHP, CTD-ILD and controls, while in IPF there was a higher proportion of males. Importantly, there were more smokers in the IPF group compared with healthy controls and non-IPF patients. All patients displayed similar time of symptoms before diagnosis, and showed a restrictive functional pattern with hypoxemia at rest worsening with exercise. However, no differences were found among the three groups of patients.

**Table 1:** Demographic and pulmonary function test variables.

	IPF (n = 85)	cHP (n = 85)	CTD-ILD (n = 85)	Control (n = 30)	p
**Age** (years)	66 ± 7	51 ± 12	57 ± 10	65 ± 10	0.4
**Gender** (Female:Male)	14: 71	81: 4	64: 21	21: 9	NA
**Time of symptoms** (months)	20 ± 17	21 ± 20	26 ± 24	NA	0.8
**Smoking status** (%) F/N/U	46/30/9	18/63/4	33/45/7	0/0/30	<0.05
**FVC** (Lts)	2.4 ± 0.7	1.7 ± 0.9	1.7 ± 0.7	3 ± 0.9	<0.001
**FVC** (%)	72 ± 21	60 ± 20	59 ± 24	90 ± 14	<0.001
**TLC** (%)	65 ± 3	61 ± 17	61 ± 19	NA	0.09
**DLCO** (%)	48 ± 25	42 ± 26	42 ± 26	NA	0.14
**Pa02**	52 ± 11	51 ± 10	54 ± 10	NA	0.2
**Rest SO2** (%)	86 ± 7	85 ± 9	86 ± 7	95 ± 3	0.6
**6 MWD: Exercise SO2** (%)	76 ± 9	73 ± 12	78 ± 10	95 ± 4	0.4
**Walking distance** (mts)	299 ± 44	380 ±125	308 ±152	482 ±176	0.9

F/N/U: former/never/unknown, FVC: forced vital capacity, TLC total lung capacity, DL_CO:_ Lung diffusion of carbon monoxide, paO2: arterial pressure of oxygen, SO_2_: saturation, 6MWD: 6-minute walk distance.

### Serum CC16 is increased in IPF patients

**Fig 1:**
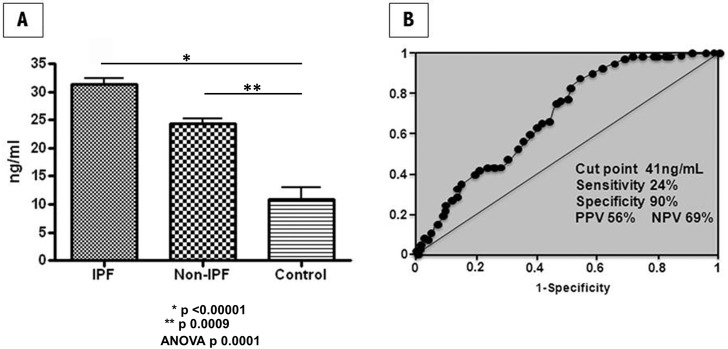
Panel A: Serum levels of CC16 are increased in patients with IPF. The concentration of serum CC16 was measured in IPF (n = 85) and non-IPF patients [cHP (n = 85), CTD-ILD (n = 85)], and healthy controls (n = 30) using ELISA. All experiments were performed in duplicate. Panel B: Receiver operating characteristic (ROC) curves used to evaluate serum CC16 as a biomarker for IPF diagnosis.

CC16 was found significantly higher in IPF compared with non-IPF patients and healthy controls (Fig 1A, S1 Dataset). Serum levels of CC16 obtained in IPF were 31.2 ± 10.8 ng/ml versus 23.1 ± 13 ng/ml in non-IPF and 10.7 ± 7.6 ng/ml in healthy volunteers (ANOVA, p<0.0001). Serum levels of CC16 in non-IPF patients were also significantly increased compared with healthy controls (p<0.009).

To determine the potential for diagnostic use of CC16 as biomarker, we applied ROC analyses (Fig 1B). According to the ROC curve, the best cut-off value for CC16 was 41 ng/mL, which yielded 24% sensitivity and 90% specificity (66.4% overall accuracy), with positive predictive value (PPV) of 56% and negative predictive value (NPV) of 69%. The AUC for IPF versus non-IPF was 0.68 (95% CI 0.613–0.745) for CC16.

We also correlated the concentrations of CC16 with the severity of baseline FVC and DLCO, which have been associated with the prognosis of IPF. However, no significant correlations were observed (data not shown). To determine whether the increase of CC16 was independent of demographic and functional variables, a multivariate linear regression model including gender, smoking, FVC, DLCO, and oxygen saturation at rest was constructed. The increase of CC16 was independent of these variables.

### CC16 is increased in bronchoalveolar lavage fluids from IPF patients and is expressed by bronchiolar and alveolar epithelial cells

**Fig 2:**
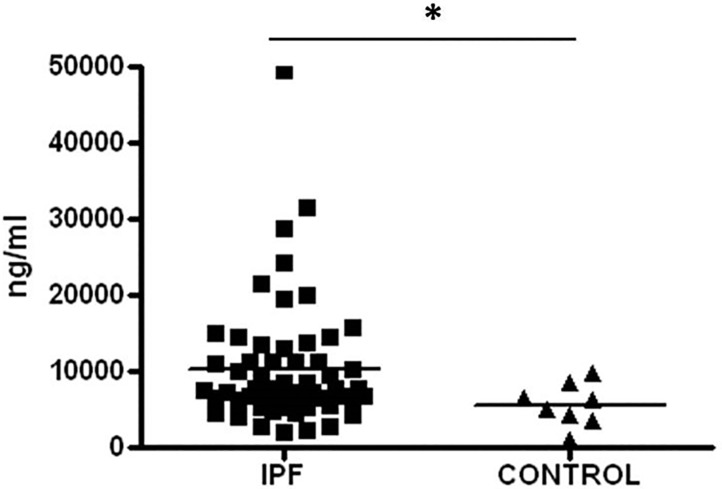
BAL concentration of CC16 is increased in patients with IPF compared with healthy controls. Data are shown as the mean ± SD. Mean values are represented by short horizontal lines. Paired comparisons were evaluated by the Mann–Whitney U-test; *p<0.03. All experiments were performed in duplicate.

As illustrated in Fig 2, BAL fluids levels of CC16 were significantly higher in IPF compared with healthy controls (10,317 ± 8,075 ng/ml versus 5622 ± 2767ng/ml; p<0.03).

**Fig 3:**
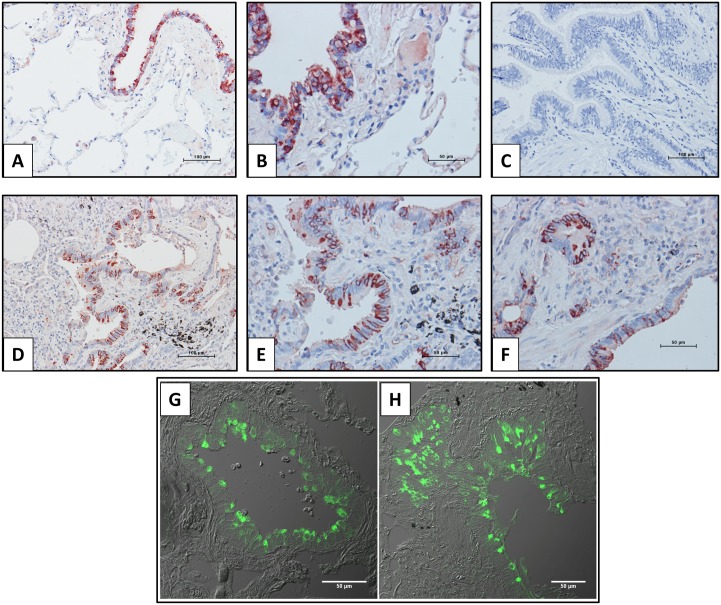
Bronchiolar localization of CC16 in IPF and control lungs. Panels A-F: Immunoreactive protein was revealed with 3-amino-9-ethyl-carbazole and samples were counterstained with hematoxylin. Panels A and B: control lungs; Panel C: negative control section from IPF lung in which the primary antibody was replaced with non-immune serum: Panels D-F: show three different IPF lungs. Panels G and H illustrate CC16 expression in bronchiolar epithelial cells revealed by immunofluorescence. 3 μm thick sections obtained from IPF (A) or healthy lung tissue (B) were treated with antibodies to detect CC10 (green).

**Fig 4:**
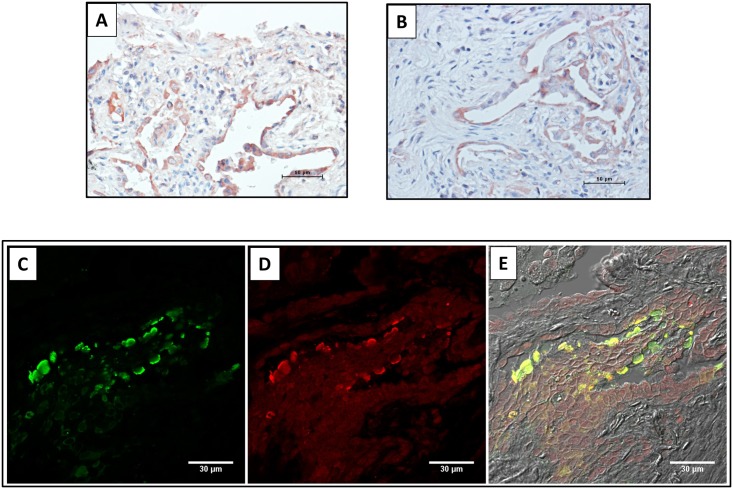
Localization of CC16 in alveolar epithelial cells. Panels A and B: IPF lung tissues illustrating positive staining for CC16 in pneumocytes. Panels C-E: Immunofluorescence double labeling for CC16 (green) and SP-C (red) demonstrated the colocalization of both proteins in the alveolar epithelium of an IPF lung (yellow).

The cellular localization of CC16 in the lungs of patients with IPF has not been previously evaluated. For this purpose, samples from IPF and normal lung tissue sections were examined by immunohistochemistry and immunofluorescence. As illustrated in Fig 3, both, control (Fig 3A and 3B) and IPF lungs (Fig 3D–3F) showed positive staining in epithelial cells from airways. No specific signal was observed when the primary antibody was replaced by non-immune serum (Fig 3C). Similar results were obtained by immunofluorescence (Fig 3G and 3H). In IPF lungs CC16 immunoreactive protein was also expressed by alveolar epithelial cells (Fig 4A and 4B), which was corroborated by colocalization with SP-C a highly specific marker of type 2 pneumocytes (Fig 4C–4E).

## Discussion

IPF is a devastating and highly lethal disease, of uncertain pathogenesis. In the last 15 years, the pathogenic paradigm for IPF has moved from one of unresolved chronic inflammation toward one of alveolar epithelial cell dysfunction that results in the over-expression of almost all the mediators responsible of fibroblast migration, proliferation and activation, and tissue remodeling [2, 20, 21].

In this study, we revealed by the first time that CC16 is also expressed by these epithelial cells in IPF lungs and that is markedly increased in serum and bronchoalveolar lavage fluids obtained from these patients. In addition, serum concentration of CC16 was significantly increased in IPF patients compared with the levels observed in non-IPF disorders, cHP and CTD-ILD, two diseases where immunopathological and inflammatory processes play a critical role.

CC16 (approved symbol SCGB1A1) is a member of the secretoglobin family of small secreted proteins mainly produced by club cells localized in bronchioles including terminal ones. It has been suggested that CC16 has anti-inflammatory and antioxidative properties in the lungs, and that might have a protective role against airways diseases like COPD and asthma [22, 23]. Actually, decreased levels of CC16 in serum is associated with reduced lung function in childhood, accelerated lung function decline in adulthood, and development of moderate airflow limitation in the general adult population [3]. Moreover, lack of CC16 substantially exacerbates cigarette smoke induced airway inflammation and alveolar loss. Likewise, lower serum CC16 concentrations are associated with excess risk of mortality, primarily by lung cancer [24]. In sharp contrast, we found that BAL and serum concentrations of CC16 are markedly increased in IPF despite that most of the studied patients were smokers (compared with non-smoker controls and significantly less smokers in the non-IPF group), a habit associated with decreased serum levels [25]. Serum concentration of CC16 was also increased in other ILD like chronic hypersensitivity pneumonitis and those associated to some connective tissue diseases, although it was significantly higher in IPF. Interestingly, higher levels of CC16 have been also reported in patients with systemic sclerosis mainly in those with active or extensive pulmonary fibrosis [26, 27]. Likewise, increase levels of serum CC16 were also recently reported in a small cohort of patients with IPF alone and IPF combined with emphysema [28].

However, our study was performed in a largest cohort of IPF patients and importantly this protein was by the first time demonstrated to be elevated in bronchoalveolar lavage suggesting a higher local production. Moreover, we also revealed that in IPF, in addition to Club cells, activated alveolar epithelial cells also contribute to its production. This is an important finding because strong evidence indicates that a dysfunctional alveolar epithelium plays a critical role in the aberrant injury/remodeling processes that occur in sporadic and familial IPF [2].

The putative role of increased production of CC16 in these disorders is presently unknown, and may be different in inflammatory-driven (cHP and CTD-ILD) and epithelial-driven fibrosis (IPF). Therefore, increased CC16 in diverse interstitial lung disorders may indicate its participation in different processes including an insufficient and overwhelm defensive mechanism or may contribute to some pathogenic mechanisms. Interestingly, it has been shown that direct contact of club cells induce apoptosis of epithelial cells from distal airways and alveoli through a tumor necrosis factor-related apoptosis-inducing ligand (TRAIL)-dependent mechanism, thus affecting wound repair [4]. However, the role of CC16 in this process, if any, was not explored. Moreover, TRAIL-expressing club cells have been localized in IPF lungs, in the proximity to the fibroblastic foci and collagen deposition and close to apoptotic alveolar epithelial cells.

However, the cause and effect of the increased CC16 as well as the remarkable difference observed between interstitial lung diseases, mainly IPF, and disorders that affect the airways, such as COPD, needs further investigation.

Another aim of our study was to evaluate whether the serum levels of CC16 may help to distinguish IPF from non-IPF interstitial diseases. The differential diagnosis of IPF with other chronic fibrosing lung disorders that exhibit an UIP pattern may be extremely difficult [1, 7, 8, 29]. In general, clinicians face two challenges for the confident diagnosis of IPF: a) the patient has IPF but the tomographic and/or morphologic findings are atypical, or b) the patient has another chronic fibrotic lung disorder with UIP-like pattern. This diagnostic uncertainty represents an important therapeutic problem since the treatment approach is completely different.

Molecular profiling analysis of blood serum/plasma has revealed several putative biomarkers, and evidence suggests that some of them, e.g., MMP-7 may help to predict IPF outcome. Recently, a biomarker index of SP-D, MMP-7, and osteopontin was found to enhance diagnostic accuracy in IPF patients compared to those with non-IPF interstitial lung diseases [14].

In general, the molecular biomarkers found so far seem to reflect the aberrant activation of alveolar epithelial cells. With this in mind, we evaluated the putative role of CC16 to distinguish IPF from non-IPF interstitial lung diseases. Our results showed that although increased levels of CC16 seems to be a risk factor to IPF, the increase of this protein by itself seems to be insufficient for differentiating IPF from non-IPF patients; however, at certain levels may increase suspicion of IPF and may complement other clinical, radiological and morphological findings to improve diagnostic accuracy.

The main limitation of this study is the lack of a replication cohort which limits our ability to draw definitive conclusions. The strengths of this study are the confident diagnosis of all the included cases, and the finding of alveolar contributions and local increase of CC16.

## Supporting Information

S1 Dataset (PDF)
