# Supplementary material for: Increased Expression of CC16 in Patients with Idiopathic Pulmonary Fibrosis
Source: PLoS One. 2016 Dec 15;11(12):e0168552. doi: 10.1371/journal.pone.0168552 (PMC5158056; doi:10.1371/journal.pone.0168552)
Supplement: S1 Dataset — (PDF) [file pone.0168552.s001.pdf]

GROUP	Gender	Age	CC16	Smoker Status
HP	Female	31	31.8	unknown
HP	Female	56	31.5	never
HP	Female	63	19.1	never
HP	Female	35	16.8	never
HP	Female	57	15.3	never
HP	Female	54	8.6	never
HP	Female	59	47.2	never
HP	Female	36	13.8	never
HP	Female	26	17.5	never
HP	Female	51	23.0	former
HP	Female	40	22.6	never
HP	Female	58	19.1	never
HP	Female	59	25.2	never
HP	Female	43	8.7	never
HP	Female	63	16.5	never
HP	Female	65	23.4	never
HP	Female	59	36.2	never
HP	Female	47	30.9	never
HP	Female	54	24.8	never
HP	Female	62	39.5	never
HP	Female	28	27.5	never
HP	Female	50	36.9	unknown
HP	Female	57	43.8	former
HP	Female	54	32.9	never
HP	Female	50	20.6	never
HP	Female	34	32.5	former
HP	Male	51	7.0	former
HP	Female	50	32.5	never
HP	Female	72	36.4	never
HP	Female	64	12.5	never
HP	Female	41	7.8	never
HP	Female	55	21.2	never
HP	Female	49	13.4	former
HP	Female	55	28.0	never
HP	Female	54	28.4	never
HP	Female	66	30.2	never
HP	Female	25	7.8	never
HP	Female	60	17.6	never
HP	Female	28	9.7	never
HP	Male	51	9.1	former
HP	Female	56	13.0	never
HP	Male	30	9.5	former

HP	Female	47	37.7	never
HP	Female	36	14.3	never
HP	Female	61	40.3	never
HP	Female	53	41.7	never
HP	Female	59	43.2	never
HP	Female	49	18.8	former
HP	Female	57	17.5	never
HP	Female	55	40.7	never
HP	Male	60	29.7	former
HP	Female	27	11.5	unknown
HP	Female	56	98.6	former
HP	Female	54	24.8	never
HP	Female	62	39.5	former
HP	Female	28	27.5	never
HP	Female	56	36.9	former
HP	Female	57	43.8	never
HP	Female	54	32.9	former
HP	Female	50	20.6	never
HP	Female	34	32.5	never
HP	Female	51	7.0	never
HP	Female	59	32.5	never
HP	Female	72	36.4	never
HP	Female	64	12.5	former
HP	Female	41	7.8	unknown
HP	Female	55	21.2	never
HP	Female	49	13.4	never
HP	Female	55	28.0	never
HP	Female	54	28.4	former
HP	Female	66	30.2	never
HP	Female	25	7.8	former
HP	Female	60	17.6	never
HP	Female	28	9.7	never
HP	Female	51	9.1	never
HP	Female	56	13.0	never
HP	Female	34	9.5	never
HP	Female	47	37.7	former
HP	Female	36	14.3	never
HP	Female	61	40.3	never
HP	Female	53	41.7	never
HP	Female	59	43.2	never
HP	Female	52	18.8	never
HP	Female	57	17.5	former
HP	Female	60	15.3	never

	Prom	50.56470588	24.9	
	DE	11.72649326	14.1	
CTD-ILD	Female	47	36.4	never
CTD-ILD	Female	45	11.5	never
CTD-ILD	Female	75	17.7	never
CTD-ILD	Female	54	14.7	unknown
CTD-ILD	Female	55	16.0	never
CTD-ILD	Female	73	28.3	never
CTD-ILD	Male	69	44.6	former
CTD-ILD	Female	49	8.2	former
CTD-ILD	Female	63	17.8	never
CTD-ILD	Female	53	20.7	never
CTD-ILD	Male	37	15.4	never
CTD-ILD	Female	61	20.5	never
CTD-ILD	Male	55	14.0	former
CTD-ILD	Female	49	25.0	unknown
CTD-ILD	Female	65	10.5	former
CTD-ILD	Female	68	12.8	never
CTD-ILD	Female	37	11.4	never
CTD-ILD	Female	30	12.1	former
CTD-ILD	Female	31	8.0	former
CTD-ILD	Male	46	54.9	never
CTD-ILD	Female	68	16.9	never
CTD-ILD	Female	61	19.2	former
CTD-ILD	Male	57	48.2	never
CTD-ILD	Female	50	19.0	unknown
CTD-ILD	Female	54	27.1	former
CTD-ILD	Female	52	3.9	former
CTD-ILD	Female	48	25.0	former
CTD-ILD	Female	59	14.8	never
CTD-ILD	Female	76	30.9	never
CTD-ILD	Female	63	36.9	never
CTD-ILD	Female	61	34.2	former
CTD-ILD	Female	54	13.8	former
CTD-ILD	Female	62	33.3	former
CTD-ILD	Male	64	13.0	unknown
CTD-ILD	Female	48	24.1	never
CTD-ILD	Female	68	17.6	never
CTD-ILD	Female	53	25.4	never
CTD-ILD	Female	68	19.8	former
CTD-ILD	Female	56	22.7	former
CTD-ILD	Male	44	13.6	never

CTD-ILD	Male	64	25.6	never
CTD-ILD	Male	60	6.6	never
CTD-ILD	Female	58	7.8	never
CTD-ILD	Female	56	10.5	never
CTD-ILD	Female	50	4.0	unknown
CTD-ILD	Female	64	19.3	former
CTD-ILD	Male	54	48.5	former
CTD-ILD	Male	64	24.3	former
CTD-ILD	Male	56	18.3	former
CTD-ILD	Female	58	33.5	former
CTD-ILD	Female	54	14.7	never
CTD-ILD	Female	55	16.0	never
CTD-ILD	Female	73	28.3	never
CTD-ILD	Male	69	44.6	former
CTD-ILD	Female	49	8.2	never
CTD-ILD	Female	63	17.8	former
CTD-ILD	Female	53	20.7	former
CTD-ILD	Male	39	15.4	former
CTD-ILD	Female	61	20.5	never
CTD-ILD	Male	55	14.0	former
CTD-ILD	Female	45	25.0	never
CTD-ILD	Female	60	10.5	never
CTD-ILD	Female	68	12.8	never
CTD-ILD	Female	39	11.4	never
CTD-ILD	Female	35	12.1	unknown
CTD-ILD	Female	33	8.0	never
CTD-ILD	Male	49	54.9	former
CTD-ILD	Female	68	16.9	former
CTD-ILD	Male	60	19.2	never
CTD-ILD	Male	57	48.2	never
CTD-ILD	Female	55	19.0	never
CTD-ILD	Female	54	27.1	never
CTD-ILD	Male	59	3.9	former
CTD-ILD	Female	50	25.0	unknown
CTD-ILD	Female	59	14.8	never
CTD-ILD	Female	76	30.9	former
CTD-ILD	Female	63	36.9	never
CTD-ILD	Female	61	34.2	never
CTD-ILD	Female	60	13.8	former
CTD-ILD	Female	62	33.3	former
CTD-ILD	Male	64	13.0	former
CTD-ILD	Female	58	24.1	never
CTD-ILD	Female	68	17.6	never

CTD-ILD	Female	58	25.4	never
CTD-ILD	Male	68	19.8	former
	Prom	56.61176471	21.4	
	DE	10.20165033	11.6	
IPF	Male	59	49.0	never
IPF	Male	73	44.7	never
IPF	Male	68	45.5	former
IPF	Male	60	27.1	former
IPF	Male	57	8.1	former
IPF	Male	60	36.1	former
IPF	Male	65	41.5	former
IPF	Female	60	18.2	former
IPF	Female	50	35.5	never
IPF	Male	58	28.4	unknown
IPF	Male	74	40.5	former
IPF	Male	70	20.1	never
IPF	Male	54	24.4	former
IPF	Female	64	29.1	former
IPF	Male	57	20.4	former
IPF	Female	70	35.7	never
IPF	Male	61	39.4	former
IPF	Male	79	24.4	former
IPF	Male	63	24.9	former
IPF	Male	70	31.5	never
IPF	Female	68	18.5	never
IPF	Male	79	38.9	never
IPF	Male	64	22.7	former
IPF	Male	70	26.2	former
IPF	Male	72	30.6	former
IPF	Male	62	30.1	former
IPF	Male	76	43.4	former
IPF	Female	66	55.7	never
IPF	Male	65	35.2	former
IPF	Male	66	21.1	former
IPF	Male	75	40.4	former
IPF	Male	57	17.5	never
IPF	Male	69	29.7	unknown
IPF	Male	76	28.3	former
IPF	Male	49	16.3	former
IPF	Female	60	25.2	never
IPF	Male	70	34.5	never
IPF	Male	58	30.3	former

IPF	Male	68	29.2	former
IPF	Male	70	26.2	unknown
IPF	Male	72	31.1	former
IPF	Male	67	21.2	never
IPF	Male	57	17.7	former
IPF	Female	70	15.7	former
IPF	Male	60	39.8	former
IPF	Female	72	48.8	former
IPF	Male	76	28.1	former
IPF	Male	67	48.3	former
IPF	Female	67	22.0	never
IPF	Male	64	27.6	former
IPF	Male	73	42.4	never
IPF	Male	76	36.9	never
IPF	Male	68	23.9	former
IPF	Male	65	60.0	former
IPF	Male	70	17.8	unknown
IPF	Male	69	61.1	never
IPF	Male	69	30.6	former
IPF	Male	79	43.3	former
IPF	Male	64	31.7	former
IPF	Male	84	44.1	former
IPF	Male	54	45.2	former
IPF	Male	70	36.4	never
IPF	Male	57	24.1	former
IPF	Male	76	30.2	former
IPF	Male	66	41.2	unknown
IPF	Male	59	24.4	former
IPF	Male	57	8.1	never
IPF	Male	60	36.1	never
IPF	Male	65	41.5	never
IPF	Female	60	18.2	never
IPF	Female	50	35.5	never
IPF	Male	58	28.4	never
IPF	Male	74	40.5	never
IPF	Male	70	20.1	never
IPF	Male	54	24.4	never
IPF	Female	64	29.1	never
IPF	Male	57	20.4	unknown
IPF	Male	70	35.7	unknown
IPF	Male	61	39.4	never
IPF	Male	79	24.4	unknown
IPF	Male	63	24.9	unknown

IPF	Female	70	31.5	former
IPF	Male	68	18.5	former
IPF	Male	79	38.9	former
IPF	Male	64	22.7	never
	Prom	65.95294118	31.2	
	DE	7.543375969	10.8	

Control	Male	63	5.4	unknown
Control	Female	52	15.3	unknown
Control	Female	56	26.1	unknown
Control	Female	66	30.4	unknown
Control	Female	59	10.8	unknown
Control	Female	66	5.4	unknown
Control	Male	74	6.3	unknown
Control	Female	53	14.0	unknown
Control	Female	87	8.6	unknown
Control	Male	75	6.7	unknown
Control	Male	74	10.8	unknown
Control	Female	73	4.9	unknown
Control	Female	67	6.0	unknown
Control	Female	73	4.5	unknown
Control	Female	66	8.7	unknown
Control	Female	69	6.3	unknown
Control	Female	50	14.0	unknown
Control	Female	50	8.6	unknown
Control	Male	53	6.7	unknown
Control	Female	56	10.8	unknown
Control	Male	72	4.9	unknown
Control	Female	58	6.0	unknown
Control	Male	66	4.5	unknown
Control	Female	59	8.7	unknown
Control	Female	77	5.4	unknown
Control	Female	61	15.3	unknown
Control	Male	64	26.1	unknown
Control	Female	50	30.4	unknown
Control	Male	64	10.8	unknown
Control	Female	67	5.4	unknown
Control	Female	91	4.2	unknown
		64.87096774	10.70709677	
		10.34002558	7.619448433	
